# ATP-Binding Cassette Genes Genotype and Expression: A Potential Association with Pancreatic Cancer Development and Chemoresistance?

**DOI:** 10.1155/2014/414931

**Published:** 2014-05-05

**Authors:** Li Pang, Beverly Word, Joshua Xu, Honggang Wang, George Hammons, Shiew-Mei Huang, Beverly Lyn-Cook

**Affiliations:** ^1^Division of Biochemical Toxicology, National Center for Toxicological Research, Food and Drug Administration, Jefferson, AR 72079, USA; ^2^Division of Bioinformatics and Biostatistics, National Center for Toxicological Research, Food and Drug Administration, Jefferson, AR 72079, USA; ^3^Office of Clinical Pharmacology, Center for Drug Evaluation Research, Food and Drug Administration, Silver Spring, MD 20993, USA

## Abstract

Genetic polymorphisms in ABC (ATP-binding cassette) transporter genes are associated with differential responses to chemotherapy in various cancers including pancreatic cancer. In this study, four SNPs in the ABCB1, ABCC1, and ABCG2 genes were investigated in normal and pancreatic cancerous specimens. The expression of the three transporters was also analyzed. The TT genotypes of G2677T and C3435T in ABCB1 gene were associated with lower risk of developing pancreatic cancer (*P* = 0.013, OR = 0.35 and *P* = 0.015, OR = 0.29, resp.). To our knowledge, this is the first report of the common polymorphisms in the ABCB1 gene affecting the genetic risk of developing pancreatic cancer. Moreover, the expression of ABCB1 in 2677TT and 3435TT carriers was lower compared to the wild-type homozygotes and heterozygotes. A cell viability assay, using standard pancreatic cancer cell lines, revealed that the ABCB1 2677TT-3455TT haplotype was more sensitive than the other haplotypes to gemcitabine. *Conclusion*. Polymorphisms in ABCB1 G2677T and G3435T were associated with differential susceptibility to pancreatic cancer and may predict responses to chemotherapy.

## 1. Introduction

Pancreatic cancer is the 10th most commonly diagnosed cancer and the 4th leading cause of cancer death in the US [1, 2]. Due to the lack of symptoms and early detection measures, pancreatic cancer is typically diagnosed at a late stage; only 10% to 15% of patients are diagnosed at a relative early stage, when surgical removal of tumor remains possible. However, because of the aggressive nature of pancreatic cancer, the recurrence rate remains very high. For up to 80% of postoperative pancreatic cancer patients, cancer reoccurs within two years after surgery. Chemotherapy is the main treatment for locally advanced, metastatic, and recurrent pancreatic cancer, but the efficacy is limited [3]. Even with gemcitabine, the golden standard for advanced pancreatic cancer treatment, the objective tumor response rate is only about 15–20% and the median survival in randomized trials is only 5–6.7 months [3]. In fact, pancreatic cancer has the highest mortality rate of all the major cancers—only 5% of patients will survive for more than five years, and the survival rate has not improved in nearly 40 years [1]. Moreover, pancreatic cancer incidence is expected to increase due to demographic changes and a number of lifestyle factors, specifically smoking, added sweeteners, and eating diets heavy in animal products [4, 5]. Understanding pancreatic cancer susceptibility and mechanism(s) of limited efficacy of chemotherapy is critical to the fight against this deadly disease.

One major reason for the limited efficacy of chemotherapy for pancreatic cancer is chemoresistance. Overexpression of ATP-binding cassette (ABC) transporters has been documented to play an important role in the development of chemoresistance in various cancers [6–8]. ABC transporters represent a superfamily of membrane proteins that actively transport a wide variety of substrates across extra- and intracellular membranes, including metabolic products, lipids, and drugs. Overexpression of ABC transporters leads to increased drug efflux thereby reducing intracellular drug levels and causing drug resistance. Of the 49 human ABC transporters, 15 are implicated in conferring resistance to chemotherapeutic agents in various cancers, and the most intensively characterized members are multidrug resistance 1 (MDR1 or P-glycoprotein, ABCB1), multidrug resistance protein 1 (MRP1, ABCC1), and breast cancer resistance protein (BCRP, ABCG2) [7]. Elevated expression of ABCB1, ABCG2, and ABCCs mRNA in pancreatic adenocarcinomas compared to normal pancreas has been reported [9–12], but correlation with clinical aggressiveness of the tumor remains controversial [11, 12].

Polymorphisms in ABC transporters have been intensively investigated and linked with varied expression of efflux pumps in different tissue compartments, altered drug levels, and host susceptibilities to several diseases. For pancreatic cancer, a recent study in pancreatic cancer survivors found that a single nucleotide polymorphism (SNP) in ABCG2 (rs2231164) correlated with pancreatic cancer survival; patients carrying the AG/GG genotypes exhibited better survival than those carrying the AA genotype [13]. Another study in patients who were treated with gemcitabine before surgery showed that two SNPs in ABCC2 and ABCC5 were associated with overall survival, and the ABCC2 G40A GG genotype was associated with poor histological response to gemcitabine [14]. The SNP ABCB1-G2677T was also reported to correlate with drug response in patients receiving adjuvant chemotherapy with gemcitabine [15]. However, very few studies have investigated the role of ABC transporter gene polymorphisms in pancreatic cancer development and whether ABC transporter genotypes are correlated with the expression of transporters in pancreas. In this study, the role of ABC transporters in pancreatic cancer development and chemoresistance was investigated. Specifically, the genetic polymorphisms of ABCB1, ABCC1, and ABCG2 in both normal and pancreatic cancerous specimens were analyzed. Expression levels of the efflux pumps were examined and correlated to the SNPs. The potential correlation of ABC transporter genotype with chemotherapy sensitivity was also investigated in several pancreatic cancer cell lines.

## 2. Materials and Methods

### 2.1. Patient Information and Sample Collection

Frozen pancreatic resection specimens were purchased from the US Cooperative Tissue Network (CHTN) (Birmingham, AL, USA) with nonidentifiable codes. The pathology and clinical information of the purchased samples were retrospectively collected from specimen information sheets. For genotyping analysis, a total of 152 samples (one pancreatic resection sample per patient) were included in the study and 120 were used for gene expression analysis. Half of the samples were from normal pancreatic tissue (NOR), and the other half were from malignant pancreatic cancerous specimens (MAL). More than 75% of the samples were from European Americans, and others were from either African Americans or ethnic unknowns (Tables 1(a) and 1(b)). For the malignant samples, the majority of them (90%) are diagnosed as pancreatic adenocarcinoma.

### 2.2. DNA Extraction and Genotyping

Four SNPs, rs2032582 (ABCB1 G2677T), rs1045642 (ABCB1 C3435T), rs4148330 (ABCC1 G-260A), and rs2231142 (ABCG2 C421T), were selected according to the following criteria: a minor allele frequency of the SNP greater than 0.1 in dbSNP and the SNP had been reported to affect expression/function of the ABC transporter or had been associated with cancer risk/clinical outcome in prior studies. Genomic DNA was extracted from pancreatic tissues or cells using QIAamp DNA kits (Qiagen, Valencia, CA, USA). Polymorphisms were detected with TaqMan SNP genotyping assays on a 7900HT Fast Real-Time PCR machine (Applied Biosystems/Life Technologies, Grand Island, NY, USA). The allelic discrimination analysis was verified with the real-time PCR results to ensure the genotyping accuracy.

### 2.3. RNA Isolation and Real-Time qRT-PCR

Total RNA was isolated from frozen pancreatic tissues or cells using RNeasy kits (Qiagen) and reverse transcribed into cDNA using RT² First Strand kits (SABiosciences/Qiagen). SYBR green-based real-time qRT-PCR was performed on the CFX96 real-time PCR detection system (Bio-Rad, Hercules, NC, USA) with gene-specific primers purchased from SABiosciences. The PCR products were verified by gel electrophoresis and melting curve analysis. The housekeeping gene phosphomannomutase 1 (PMM1) was used as the endogenous standard for normalization because, as others had reported [16, 17], we found that the expression of GAPDH, but not PMM1, was increased in pancreatic cancerous specimens.

### 2.4. Cell Culture and Cell Viability Assay

The human pancreatic cancer cells, BXPC-3, AsPC-1, CFPAC-1, PANC-1, PL-45, MiaPaca-2, and SU86.86, were obtained from the American Type Culture Collection (ATCC) (Manassas, VA, USA) and cultured as previously described [18]. Briefly, 24 hours after seeding (in 96-well plates), the pancreatic cancer cells were treated with different concentrations of gemcitabine (Eli Lilly Co., Indianapolis, IN, USA) for 48 hours and the cell viability was determined with the CellTiter 96 AQueous One Solution Cell Proliferation assay (Promega, Madison, WI, USA) by following the manufacturer's instructions. The concentration of gemcitabine required to cause 50% growth inhibition (IC_50_) was calculated using Graphpad Prism 6 software (San Diego, CA, USA).

### 2.5. Statistical Analysis

The genotyping data were analyzed with PLINK (http://pngu.mgh.harvard.edu/purcell/plink/). The independence of genotype frequencies of the studied SNPs was tested for Hardy-Weinberg equilibrium. Differences in the frequencies of the ABCB1, ABCC1, and ABCG2 gene polymorphisms between NOR and MAL specimens were analyzed using Fisher's Exact tests. Odds ratios (ORs) and 95% confidence intervals (CIs) were calculated for the allelic and genotypic comparisons, following codominant, dominant, and recessive genetic model tests. Unless stated otherwise, the gene expression data were compared using an unpaired *t*-test. In all instances, results were considered statistically significant at the level of *P* < 0.05.

## 3. Results

In the total number of 152 samples (121 European Americans) analyzed in this study, the distribution of genotypic and allelic frequencies of all four SNPs met the Hardy-Weinberg equilibrium in both the whole study population and the European American subgroup. Although the allele frequencies of the four SNPs did not differ between the NOR and MAL groups in the whole study population, the mutant allele T in rs2032582 (ABCB1 2677T) tended to have a higher frequency in NOR than MAL European Americans (*P* = 0.057, data not shown). For the genotypic frequencies, the mutant homozygous genotypes of rs2032582 and rs1045642 (2677TT and 3435TT) in the ABCB1 gene were significantly associated with reduced risks of developing pancreatic cancer in the whole study population and the European American subgroup (Tables 2 and 3, *P* = 0.015, OR = 0.29 and *P* = 0.013, OR = 0.35, resp., for the whole study population; *P* = 0.043, OR = 0.34 and *P* = 0.033, OR = 0.39, resp., for the European Americans). The distributions of the two SNPs were also statistically significant for the recessive genetic model testing both in the whole study population and in the European American subgroup (*P* = 0.014 and 0.049 for G2677T; *P* = 0.017 and 0.035, for C3435T, resp.). No statistical differences were found for the allele and genotype frequencies of the other two SNPs between NOR and MAL pancreatic samples.

**Figure 1:**
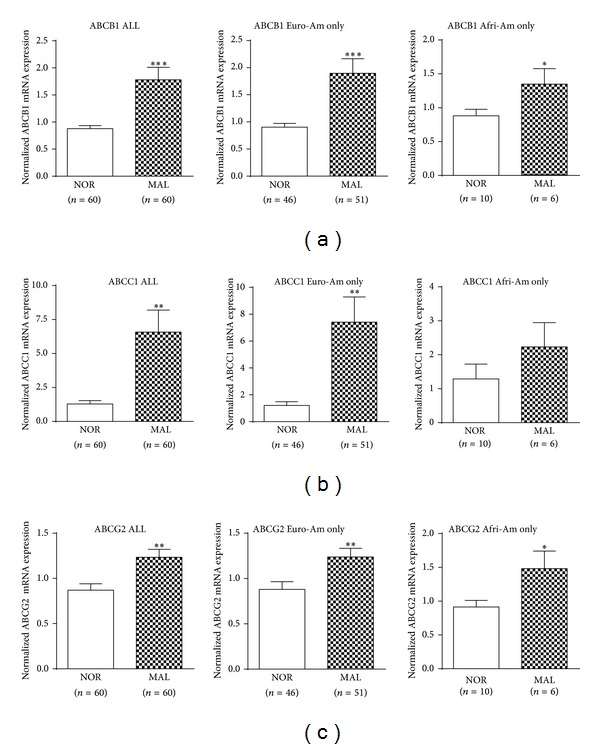
Comparison of ABCB1, ABCC1, and ABCG2 gene expression in normal and pancreatic cancerous tissue. The individual expression levels of the indicated ABC transporters (a–c) were analyzed by real-time qRT-PCR, normalized to mRNA expression of PMM1, and reported as x-fold relative to the expression of a calibrator (a premixed pancreatic RNA sample, set as 1). Values are expressed as means ± SEM, *P < 0.05, **P < 0.01, and ***P < 0.001. Euro-Am, European Americans; Afri-Am, African Americans.

**Figure 2:**
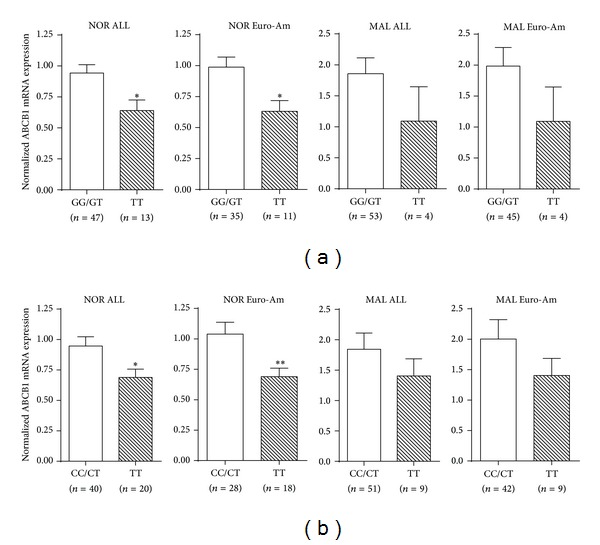
Expression of ABCB1 based on genotypes. The mRNA expression of ABCB1 in mutant homozygotes of G2677T (a) and C3435T (b) was compared to the wild-type homozygotes and heterozygotes in both whole study population (ALL) and the European Americans (Euro-Am). Values are expressed as means ± SEM, *P < 0.05, and **P < 0.01.

Expression of ABCB1, ABCC1, and ABCG2 genes was analyzed and all three ABC transporters were found to be significantly increased in MAL specimens compared to NOR pancreases in almost all the study populations/subgroups, except for the expression of ABCC1 in African Americans (Figure 1). Expression of the three ABC transporters was also compared based on their genotypes and no correlations of mRNA expression with genotypes in ABCC1 G-260A and ABCG2 C421T were found (data not shown). However, compared to other genotypes, the mutant homozygotes of ABCB1 2677TT and 3435TT were correlated with significantly reduced expression of ABCB1 in NOR pancreases in both the whole study population and European American subgroup (Figures 2(a) and 2(b)). Similar trends were also seen in the MAL specimens, but the differences were not statistically significant.

**Figure 3:**
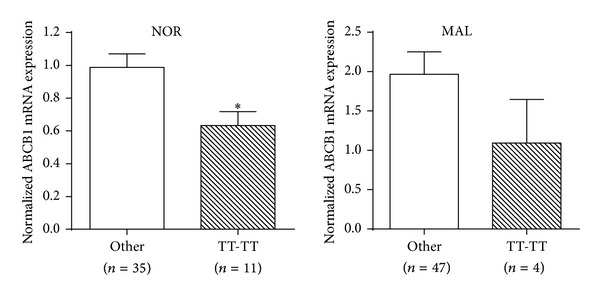
Expression of ABCB1 based on G2677T-C3435T haplotypes. The mRNA expression of ABCB1 in carriers of 2677TT-3435TT haplotype was compared to those of all the other haplotypes in European Americans. Values are expressed as means ± SEM and *P < 0.05.

Because the ABCB1 G2677T and C3435T were in linkage disequilibrium, the haplotype frequencies of the two SNPs were also compared between NOR and MAL specimens and correlated with mRNA expression in European Americans. The ABCB1 2677TT-3435TT haplotype was significantly associated with reduced risk of developing pancreatic cancer (OR 0.27, 95% CI 0.08 to 0.92, *P* = 0.037) in European Americans, and the expression of ABCB1 was also lower compared to the other haplotypes (Figure 3).

Pancreatic cancer cell lines are commonly used to study the mechanisms of chemoresistance. To investigate whether the protective ABCB1 genotype/haplotype identified in this study was correlated with any functional significance in response to chemotherapy drugs, we analyzed the expression of the ABCB1 gene, the genetic polymorphism of ABCB1, and the sensitivity to gemcitabine in seven pancreatic cancer cell lines. Interestingly, three of the cell lines, MiaPaca-2, BXPC-3, and CFPAC-1, were found to be ABCB1 2677TT and 3435TT homozygotes. The cell viability assay showed that these three cell lines were more sensitive to gemcitabine than PANC-1, SU86.86, PL-45, and AsPC-1, which were either ABCB1 G2677T-C3435T wild-type homozygotes or heterozygotes (Table 4). However, there was no association between ABCB1 mRNA expression and sensitivities to gemcitabine in these cell lines (Table 4).

## 4. Discussion

Many factors have been linked to the increased risks of developing pancreatic cancer, including age, race, obesity, cigarette smoking, diabetes, chronic pancreatitis, and genetic factors. Indeed, genetic factors play an important role in both familial and sporadic occurrences of pancreatic cancer. Mutations in the high penetrance genes such as BRCA2, PALB2, p16/CDKN2A, PRSS1, SPINK1, and STK11 correlate with a very high lifetime risk of developing pancreatic cancer and may cause as many as 10% of pancreatic cancers in the US [19]. For the nonfamilial (sporadic) pancreatic cancer, a few low penetrance genes have been identified by genome-wide association studies (GWAS). Pancreatic cancer candidate genes have also been reported from studies that examined the biological pathways known to be important in the development of pancreatic cancer, for example, tobacco metabolism, DNA repair, inflammation, and folate metabolism [19, 20].

Genetic variants in ABC transporter genes have been intensively investigated and linked to differential disease susceptibilities and varied responses to therapeutic drugs [7]. In this study, the ABCB1 2677TT and 3435TT genotypes were found to be associated with reduced risk of developing pancreatic cancer in the whole study population (Table 2) and European Americans (Table 3). In fact, combination of the data of European Americans with the ethnic unknowns (considering more than 80% of pancreatic cancer patients in the US are European Americans) showed that the distribution of the ABCB1 G2677T was statistically significant for four of the PLINK tests, including Fisher's Exact test for allelic and genotypic frequencies (*P* = 0.043 and 0.039, resp.), Cochran-Armitage trend test (*P* = 0.029), and recessive genetic model test (*P* = 0.020); for ABCB1 C3435T, Fisher's Exact test for genotypic frequency and recessive genetic model test were statistically significant (*P* = 0.028 and *P* = 0.013, resp.); the *P* values of Cochran-Armitage trend test (*P* = 0.058) and Fisher's Exact test for allelic frequency (*P* = 0.066) were close to statistical significance. Due to the smaller sample size, we could not test the association in African Americans. Even with additional samples, it would remain very difficult to obtain sufficient statistical power to detect the association because of the very low genotype frequencies of ABCB1 2677TT and 3435TT in African Americans [21–23]. To our knowledge, this is the first report of the common polymorphisms in the ABCB1 gene affecting the genetic risk of developing pancreatic cancer. More independent studies with larger sample sizes are needed to verify this interesting discovery.

ABCB1, ABCC1, and ABCG2 gene expression was significantly increased in the MAL specimens compared to NOR pancreases (Figure 1). This finding is consistent with other reports [9–12] for the potential involvement of ABC transporters in pancreatic cancer chemoresistance.

The ABCB1 2677TT and 3435TT genotypes/haplotypes were associated with reduced expression of ABCB1 in normal pancreatic tissue (Figures 2 and 3), although the differences were not statistically significant in pancreatic cancerous specimens, which might be because of the smaller sample size of TT variants in MAL group. In fact, this result is consistent with our observation that fewer carriers of ABCB1 2677TT and 3435TT developed pancreatic cancer (Tables 2 and 3). The nonsynonymous mutation in ABCB1 G2677T can result in a distinct amino acid change (Ala > Ser), which exhibited lower substrate specificity and reduced drug-stimulated ATPase activity as compared to the wild type [23]. The ABCB1 C3435T is a synonymous mutation but the variant can alter protein expression by affecting translation efficacy [24]. Since ABCB1 3435TT was first reported to be significantly associated with reduced ABCB1 expression in intestine compared to the CC homozygotes, numerous studies have investigated the association of ABCB1 genotype/haplotype with expression/function of the transporter in different tissues [25]. However, the results were not consistent. Our study is the first to compare the expression of ABC transporters in pancreatic tissue based on genotypes. Clearly, additional studies are necessary to confirm the results from this study.

Gemcitabine is commonly used as an adjuvant therapy for the treatment of postoperative pancreatic cancer and remains the standard of care for advanced pancreatic cancer, despite the very low patient response rate due to innate and acquired chemoresistance. While the human equilibrative transporters have been identified to be able to mediate gemcitabine uptake, the role of efflux pumps in gemcitabine transport has not yet been clearly delineated. Deoxycytidine kinase is the rate-limiting enzyme for gemcitabine activation and cytidine deaminase inactivates gemcitabine to difluorodeoxyuridine, allowing the metabolite to be cleared from the cell [26]. Interestingly, 2′,2′-difluorodeoxyuridine (dFdU), the major inactive metabolite of gemcitabine, is a substrate of ABC transporter [27]. Nonselectively inhibiting ABC transporters' activity could significantly increase intracellular dFdU level, inhibit cytidine deaminase, and result in an increase of intracellular gemcitabine concentration and enhanced cytotoxicity [27]. Although gemcitabine may not be a direct substrate of ABCB1, changes in expression or function of ABCB1 can be contributed to the development of gemcitabine chemoresistance. Due to high levels of ABCB1 gene expression being linked to poor prognosis of human pancreatic cancer [11] and the fact that ABCB1 2677TT and 3435TT genotypes were reported to be associated with increased overall survivals in gemcitabine treated postoperative pancreatic cancer patients [15], this study examined the association of ABCB1 genotype/haplotype with ABCB1 mRNA expression and the sensitivity to gemcitabine in several pancreatic cancer cell lines. Interestingly, we found that the cell lines with the ABCB1 2677TT-3435TT haplotype were more sensitive to gemcitabine than the cells carrying the other haplotypes (Table 4) but that the differences could not be explained by the varied expression of ABCB1 nor by the known mutations found in these cell lines [28]. The ABCB1 2677-3435 haplotypes in these pancreatic cancer cell lines might be associated with other unknown mechanisms that affect the cells' sensitivity to gemcitabine.

ABC transporters are not just drug efflux pumps. Many studies have elucidated the additional roles of ABC transporters in cancer initiation and progression [8]: for example, (1) ABCB1 has been reported to inhibit the apoptotic cascade in both normal and cancer cells; (2) knockdown of ABCB1 by small interfering RNA suppressed cancer cell proliferation and tumor expansion in a mouse xenograft model; (3) ABCB1 has also been reported to play a role in cell proliferation and delivering of protumorigenic platelet activating factor to its receptor. Any of these mechanisms could possibly explain the striking finding from this study that lower expression levels of ABCB1 in the normal 2677TT and 3435TT carriers are associated with reduced risk of developing pancreatic cancer.

ABCB1 G2677T and C3435T are two functional SNPs with varied frequencies in different populations [21, 22, 25]. The very low frequencies of 2677TT and 3435TT genotypes/haplotypes in African American populations may partially explain why African Americans are more frequently affected by pancreatic cancer than European Americans and Asian Americans [29]. African Americans have the highest incidence and mortality rates of pancreatic cancer compared to the other ethnic groups in the US [29]. The lack of protective genotype/haplotype, ABCB1 2677TT and 3435TT, may contribute to a higher susceptibility of African Americans to pancreatic cancer and increased likelihood of gemcitabine chemoresistance, thus poor prognosis.

In conclusion, this study has found that (1) the ABCB1 2677TT and 3435TT genotypes/haplotypes are associated with lower risk of developing pancreatic cancer; (2) the mRNA expression of ABCB1 reduced in the ABCB1 2677TT and 3435TT carriers; and (3) the ABCB1 2677TT-3455TT haplotype might be linked to an increased sensitivity to gemcitabine compared to the other haplotypes. The results from this study may aid in the future practice of utilizing pharmacogenomics to guide pancreatic cancer chemotherapy. However, large studies in different ethnic groups are needed to further confirm these findings.

## Figures and Tables

**Table:** (a)

	Age (yr)	Sex *n* (%)	Race *n* (%)
NOR (76)	56.8 ± 1.9	F 41 (53.9) M 35 (46.1)	Euro-Am 58 (76.3) Afri-Am 12 (15.8)
MAL (76)	65.1 ± 1.5	F 39 (52.0) M 36 (48.0)	Euro-Am 63 (82.9) Afri-Am 8 (10.5)

**Table:** (b)

	Age (yr)	Sex *n* (%)	Race *n* (%)
NOR (60)	57.0 ± 2.1	F 33 (55.0) M 27 (45.0)	Euro-Am 46 (76.7) Afri-Am 10 (16.7)
MAL (60)	64.1 ± 2.0	F 32 (53.3) M 27 (45.0)	Euro-Am 51 (85.0) Afri-Am 6 (10.0)

**Table 2:** Genotypic frequency in the whole study population.

Genotypes	NOR (*n* = 76) *N* (freq.%)	MAL (*n* = 76) *N* (freq.%)	OR (95% CI)	*P* value
ABCB1 G2677T/A			0.29 (0.11∼0.79)	0.015
GG	27 (35.5)	28 (36.8)	TT versus (GT+GG)	
GT	31 (40.8)	38 (50.0)		
TT	18 (23.7)	6 (7.9)		
TA	0 (0)	2 (2.6)		
GA	0 (0)	2 (2.6)		
ABCB1 C3435T			0.35 (0.15∼0.80)	0.013
CC	22 (28.9)	21 (27.6)	TT versus (CT+CC)	
CT	31 (40.8)	45 (59.2)		
TT	23 (30.3)	10 (13.2)		
ABCC1 G-260A			1.49 (0.62∼3.60)	0.376
GG	10 (13.2)	14 (18.4)	GG versus (GA+AA)	
GA	35 (46.0)	32 (42.1)		
AA	31 (40.8)	30 (39.5)		
ABCG2 C421T			5.13 (0.24∼108.70)	0.294
CC	61 (80.3)	62 (81.6)	TT versus (CT+CC)	
CT	15 (19.7)	12 (15.8)		
TT	0 (0)	2 (2.6)		

**Table 3:** Genotypic frequency in European Americans.

Genotypes	NOR (*n* = 58) *N* (freq.%)	MAL (*n* = 63) *N* (freq.%)	OR (95% CI)	*P* value
ABCB1 G2677T/A			0.34 (0.12∼0.97)	0.043
GG	16 (27.6)	23 (36.5)	TT versus (GT+GG)	
GT	28 (48.3)	32 (50.8)		
TT	14 (24.1)	6 (9.5)		
TA	0 (0)	1 (1.6)		
GA	0 (0)	1 (1.6)		
ABCB1 C3435T			0.39 (0.16∼0.92)	0.033
CC	13 (22.4)	16 (25.4)	TT versus (CT+CC)	
CT	26 (44.8)	37 (58.7)		
TT	19 (32.8)	10 (15.9)		
ABCC1 G-260A			1.96 (0.56–6.91)	0.293
GG	4 (6.9)	8 (12.7)	GG versus (GA+AA)	
GA	29 (50.0)	27 (42.9)		
AA	25 (43.1)	28 (44.4)		
ABCG2 C421T			2.81 (0.11–70.31)	0.530
CC	45 (77.6)	52 (82.5)	TT versus (CT+CC)	
CT	13 (22.4)	10 (15.9)		
TT	0 (0)	1 (1.6)		

**Table 4:** Pancreatic cancer cell line ABCB1 mRNA expression, haplotype, and sensitivity to gemcitabine.

Cell line	ABCB1 expression	ABCB1 haplotype	GEM IC_50_ (*μ*M)
AsPC-1	24.67	2677GG-3435CC	25.59
SU86.86	0.34	2677GG-3435CC	>100
PL-45	0.98	2677GG-3435CT	18.75
PANC-1	1	2677GT-3435TT	>100
BXPC-3	15.82	2677TT-3435TT	0.11
MiaPaca-2	0.75	2677TT-3435TT	1.90
CFPAC-1	1015.7	2677TT-3134TT	1.09

GEM: gemcitabine. The expression of ABCB1 gene is normalized to PMM1 and showed as x-fold relative to the expression in PANC-1 cells.
